# Investigating the Promising P28 Peptide-Loaded Chitosan/Ceramic Bone Scaffolds for Bone Regeneration

**DOI:** 10.3390/molecules29174208

**Published:** 2024-09-05

**Authors:** Keran Zhou, Bianca Simonassi-Paiva, Gustavo Fehrenbach, Guangming Yan, Alexandre Portela, Robert Pogue, Zhi Cao, Margaret Brennan Fournet, Declan M. Devine

**Affiliations:** 1 PRISM Research Institute, Technological University of the Shannon, Midlands Midwest, Athlone Main Campus, N37 HD68 Athlone, Ireland; a00258808@student.tus.ie (K.Z.);; 2 Genomic Sciences and Biotechnology Program, Catholic University of Brasilia, Brasília 71966-700, Brazil

**Keywords:** bone regeneration, bone tissue engineering, bone scaffold, osteoblast differentiation

## Abstract

Bone has the ability to heal itself; however, bone defects fail to heal once the damage exceeds a critical size. Bone regeneration remains a significant clinical challenge, with autograft considered the ideal bone graft material due to its sufficient porosity, osteogenic cells, and biological growth factors. However, limitations to bone grafting, such as limited bone stock and high resorption rates, have led to a great deal of research into developing bone graft substitutes. The P28 peptide is a small molecule bioactive biomimetic alternative to mimic the bone morphogenetic protein 2 (BMP-2). In this study, we investigated the potential of P28-loaded hybrid scaffolds to mimic the natural bone structure for enhancing the bone regeneration process. We hypothesized that the peptide-loaded scaffolds and nude scaffolds both have the potential to promote bone healing, and the bone healing process is accelerated by the release of the peptide. To verify our hypothesis, C2C12 cells were evaluated for the presence of calcium deposits by histological stain at 7 and 14 days in cultures with hybrid scaffolds. Total RNA was isolated from C2C12 cells cultured with hybrid scaffolds for 7 and 14 days to assess osteoblast differentiation. The project findings demonstrated that the hybrid scaffold could enhance osteoblast differentiation and significantly improve the therapeutic effects of the scaffold in bone regeneration.

## 1. Introduction

Bone possesses the innate potential to repair itself; however, critical-sized defects (CSDs) and non-union require surgical intervention to promote bone regeneration [1,2,3]. CSDs can arise from bone fractures, congenital disorders, cancer resection surgeries, etc., and result in an estimated two million bone grafts annually [4]. Bone grafts are the second most commonly implanted biological material globally. These bone grafts commonly come from the patients’ non-essential bone stock (autograft), another donor (allograft), or animal sources (xenograft) [5,6]. Autografts are still the gold standard in clinical bone grafts, but they present major limitations, such as donor-site morbidity, limited bone mass, prolonged operation time, and high resorption rates [7]. Allografts and xenografts have led to a great deal of research into the development of bone graft substitutes [4,8,9]. Bone tissue engineering (BTE) has promising potential for bone graft candidates, since it combines bone graft substitute materials with biological cues similar to those present in autografts that signal bone repair [10,11]. 

Native bone is a complex structure comprised of collagenous proteins, calcium phosphate, cells, growth factors, and water. Much research in the field of BTE seeks to develop a biomimetic structure using polymer materials, ceramic, metal, and composite materials that have been utilized in BTE over the last decade [12,13]. An ideal bone scaffold should promote cell adhesion, proliferation, differentiation, provide structural support, and exhibit osteoconductivity and controllable biodegradability [14,15]. Bone scaffolds may be prepared by different methods, such as freeze-drying, fiber-bonding, gel-casting, and 3D printing/additive manufacturing, etc. [10].

Natural-derived polymers such as chitosan (CS)-based ceramic composite scaffolds have been investigated for use in BTE. CS is a basic amino polysaccharide semi-synthesized from deacetylation chitin by alkaline hydrolysis at high temperatures [16,17]. CS’s characterization meets the essential requirements of a bone scaffold, and it also facilitates the proliferation of osteoblast cells and mineralized bone matrix regeneration [18]. CS-based ceramic scaffolds have been extensively investigated for manufacturing orthopedic devices and it has been proven to exhibit antibacterial, biocompatibility, and biodegradability properties, as well as minimal foreign-body reaction [19]. Hydroxyapatite (Ca_10_(PO_4_)_3_OH) (HAp) has drawn significant attention as a bone substitute material due to its similar structure and composition to bone. In addition to its osteoconductive and osteoinductive potential, HAp also exhibits bioactivity, mechanical strength, and excellent chemical and biological affinity for bony tissue [17,20]. The major drawback of HAp is its slow degradation rate. The substitution of the hydroxyl group (OH^−^) with a fluoride ion (F^−^) in HAp can significantly increase the dissolution rate of HAp in vivo [21]. FAp is prepared when OH^−^ groups in HAp are replaced with F^−^ [22].

Scaffolds incorporated with growth factors have been shown to enhance their osteoinductive ability. Bone morphogenic protein-2 (BMP-2) plays a key role in osteoinductive growth factors [23,24,25]. However, BMP-2 poses numerous drawbacks, such as its short half-life, inflammatory reaction, bone resorption, and cancer risk. P28 is a short BMP-2-related peptide that can overcome the above-cited drawbacks [26]. In addition, the P28 peptide has a higher affinity for hydroxyapatite because of seven repeated aspartic acids linked to phosphorylated serine motifs in the P28 peptide [27,28].

In this study, a novel chitosan/hydroxyapatite/fluorapatite (CS/HAp/FAp) bone scaffold was prepared to develop the delivery system for optimizing the biological effect of P28 through freeze-drying. The highly interconnected pore is the key to transport nutrients, oxygen, cell proliferation, and new tissue regeneration. It has certain osteoinductive properties and a significant osteoconductive properties. Experiments demonstrated that the novel CS/HAp/FAp bone scaffold shows good potential for treating bone defects. Thus, the aim of this study was to elucidate whether the peptide-loaded scaffolds are more suitable as alternatives for bone defects compared to nude scaffolds.

## 2. Results

### 2.1. Scanning Electron Microscopy (SEM)

The porous structure of scaffolds can affect cellular responses, from adhesion and migration to differentiation and new-tissue regeneration [29]. The porous morphology of scaffolds is the critical parameter for the invasion of cells from the surrounding environment, contributing to vascularization [30].

**Figure 1:**
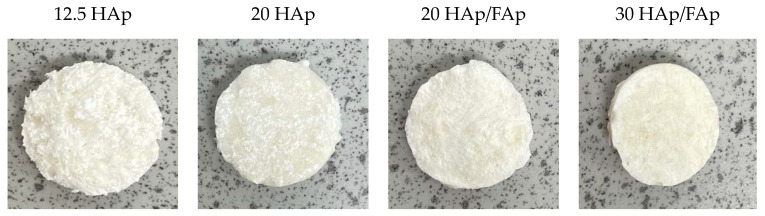
Micrographs of the hybrid scaffolds.

The macroscopic images show that there is no obvious visual difference among the scaffolds; all appeared as white, three-dimensional structures with a similar size, homogeneous texture, and porous appearance (shown in Figure 1).

**Figure 2:**
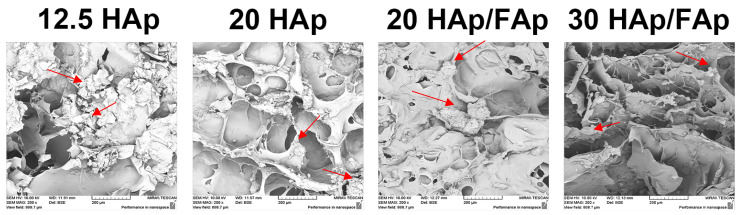
SEM micrographs of scaffolds with precipitated ceramic particles within a chitosan matrix, red arrows correspond to ceramic particles.

SEM was carried out to observe the morphology of hybrid scaffolds (as shown in Figure 2). The scaffolds revealed a non-uniform porous microstructure, and ceramic particles were distributed randomly within a smooth chitosan layer. It was indicated that the crosslinking reaction was successful [29]. Moreover, the different formulations of scaffolds were similar in microscopic morphology, which may show that ceramic will not affect the porous structure [31]. On the other hand, the bulge of the ceramic might enhance mechanical strength and provide more contact areas for cells, which could promote cell adhesion [32,33]. The increasing acetic acid volume appears to facilitate the formation of the more interconnected porous network, which benefits cell infiltration and nutrient transport during the bone regeneration period.

### 2.2. Antibacterial Activity

**Figure 3:**
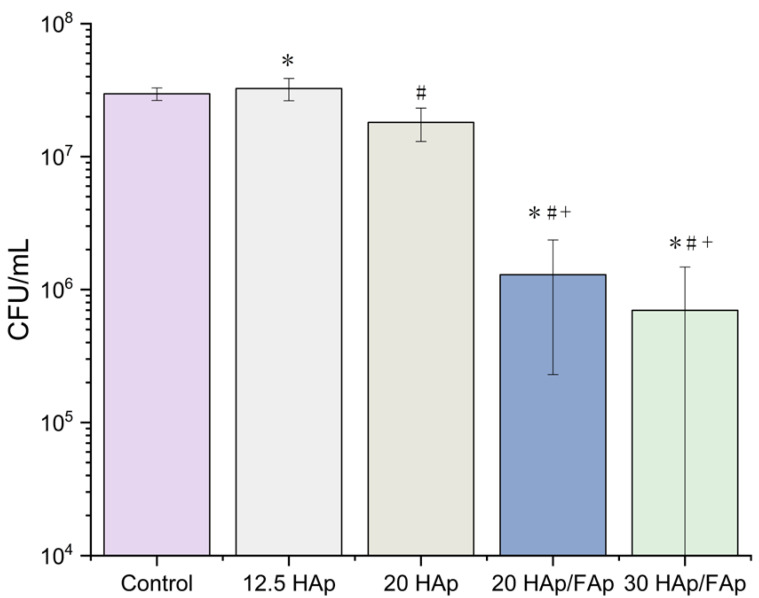
Bacteria proliferation using the flow cytometry method indicated that all scaffolds had antibacterial properties (* *p* < 0.05 vs. control, ^#^ *p* < 0.05 vs. 12 HAp, ^+^ *p* < 0.05 vs. 20 HAp).

The in vitro antibacterial activity of the hybrid scaffolds against Gram-positive Staphylococcus aureus (*S. aureus*, ATCC6538) was assessed by the flow cytometry method. Flow cytometry is used to measure the fluorescence when the bacteria go through the laser beam [34]. SYBR green binds to DNA, and because it is membrane-permeant, it can enter both live and dead cells. Viable cells, however, can be differentiated from non-stained and dead cells by using controls, such as negative controls with ethanol, which causes oxidative DNA damage, and consequently lower counts when compared to groups in the positive control [35]. The quantitative analysis of bacteria was assessed in samples collected after culturing the *S. aureus* with the hybrid scaffolds for 24 h (shown in Figure 3). The traditional treatment for infected orthopedic implants is the injection of antibiotics into the bone infection site. However, there has been an increase in antibiotic-resistant bacteria and a related rise in mortality rates in recent years [36,37]. As such, a scaffold that exhibits antibacterial properties is seen as a good strategy to inhibit bacteria colonization. In this study, FAp was employed (among other reasons) due to its reported antimicrobial functionality [21,38]. In this work it can be seen that the addition of FAp to the scaffolds resulted in the inhibition of *S aureus* following co-culture with 20 HAp/FAp scaffolds, compared with the 20 HAp scaffolds (*p* < 0.05). It was demonstrated that FAp had the potential to inhibit Gram-positive bacterial proliferation, in agreement with findings reported in other papers [39]. Furthermore, increasing the initial volume of acetic acid also affected the antibacterial properties of scaffolds: 20 HAp scaffolds had better antibacterial properties compared to 12 HAp scaffolds.

### 2.3. Cell Viability

The response of the C2C12 cells to the hybrid scaffolds was investigated by the MTT (3-(4,5-Dimethylthiazol-2-yl)-2,5-Diphenyltetrazolium Bromide) assay. However, other papers have reported that the conventional MTT assay does not produce consistent results when used to measure cellular proliferation on the scaffolds [40]. It is particularly notable in cases where scaffolds may absorb Formozan stain. To overcome this the shortcoming, an indirect MTT assay method was used to determine cell viability.

**Figure 4:**
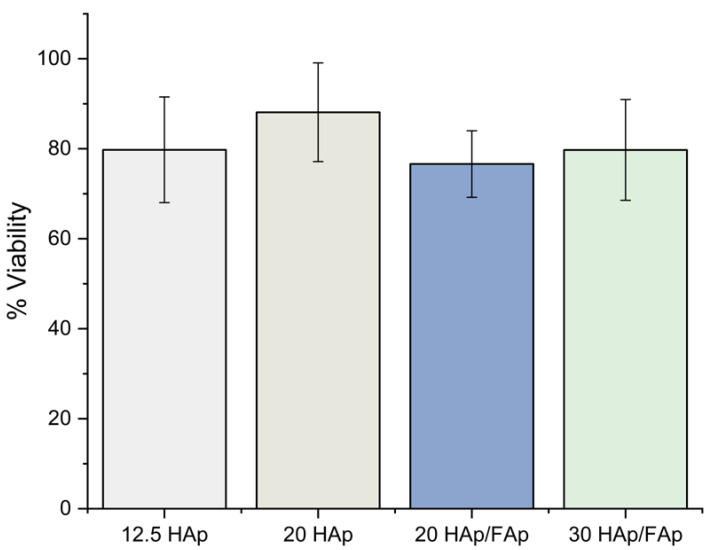
Cell viability using the indirect MTT assay method indicates that all scaffolds exhibit cytocompatibility.

Cell viability using the indirect method was greater than 70% for all scaffolds, indicating the cytocompatibility of the scaffolds. No significant differences were detected between the groups (*p* > 0.05) (shown in Figure 4).

### 2.4. Alizarin Red S Staining

**Figure 5:**
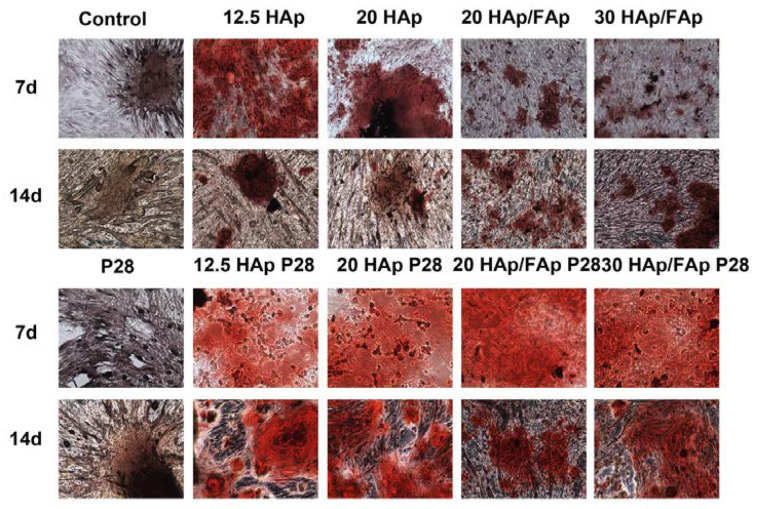
Alizarin Red S staining for C2C12 cells after culturing with scaffolds for 7 and 14 days.

C2C12 cells were seeded and cultured with scaffolds for osteogenic differentiation for 7 and 14 days. During the osteogenic differentiation stage, the mineralization phase starts with the cells secreting calcium phosphate minerals [41]. Calcium nodule deposition is one of the key biomarkers of osteogenic differentiation [42,43]. Extracellular matrix mineralization was detected by Alizarin Red S (ARS) staining, where calcium deposits are positively stained in red [44,45]. On days 7 and 14, no significant difference was found between the control group and the P28-treated group, as shown in Figure 5. Both groups only had some orange spots. However, positive nodular aggregates were already observed in the cells cultured with scaffolds at day 7, as shown under the microscope camera, but the red aggregates were significantly smaller and lighter compared to those in the cells cultured with scaffold-loaded P28 groups. It indicated better extracellular mineralization, as prompted by the P28-loaded scaffolds.

**Figure 6:**
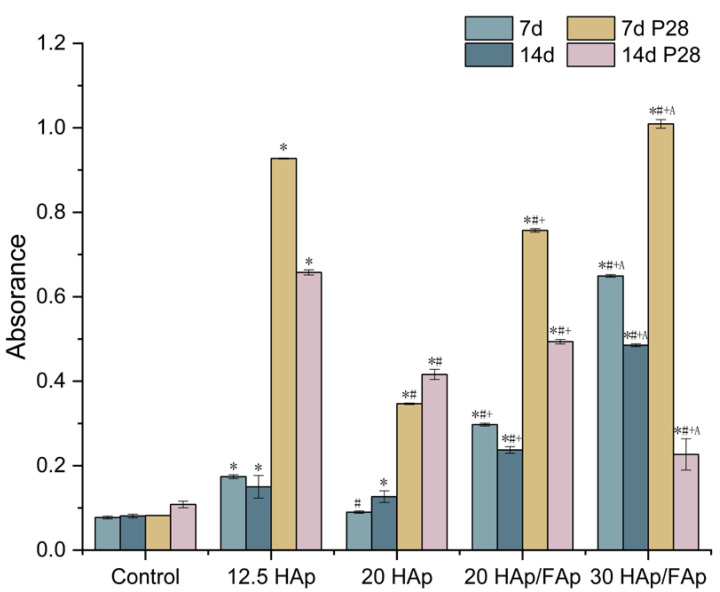
The quantification of Alizarin Red S staining for different formulations scaffolds and peptide loaded scaffolds at 7 and 14 days. (* *p* < 0.05 vs. control, ^#^ *p* < 0.05 vs. 12 HAp, ^+^ *p* < 0.05 vs. 20 HAp, ^A^ *p* < 0.05 vs. 20 HAp/Fap).

Furthermore, the quantitative analysis of ARS staining showed that the OD values exhibited a similar trend to the colorimetric quantification, as shown in Figure 6. The amount of mineralization in the control groups was lower than in the other scaffold groups (*p* < 0.05), which indicated that the scaffolds significantly facilitated calcium deposits at 7 and 14 days. 

Compared with the nude scaffold groups, the P28-loaded scaffolds showed significant differences. However, the P28-loaded 30 HAp/FAp scaffold had a smaller amount of calcium deposit compared to the nude 30 HAp/FAp scaffold at 14 days. The P28 peptide may diffuse more easily as a result of the loose stricture of 30 HAp/FAp. The findings also demonstrated that the P28-loaded 30 HAp/FAp group differed significantly from all other groups. 

Fluoride ions have been reported to promote the bone regeneration process by facilitating bone mineralization [22]. The 20 HAp/FAp group was more efficient at forming a mineralized matrix compared to the 20 HAp group (*p* < 0.05). It was demonstrated that the addition of fluorapatite may contribute to an increased calcium source, enhancing the availability of calcium ions for mineralization.

### 2.5. Alkaline Phosphatase (ALP) Activity

**Figure 7:**
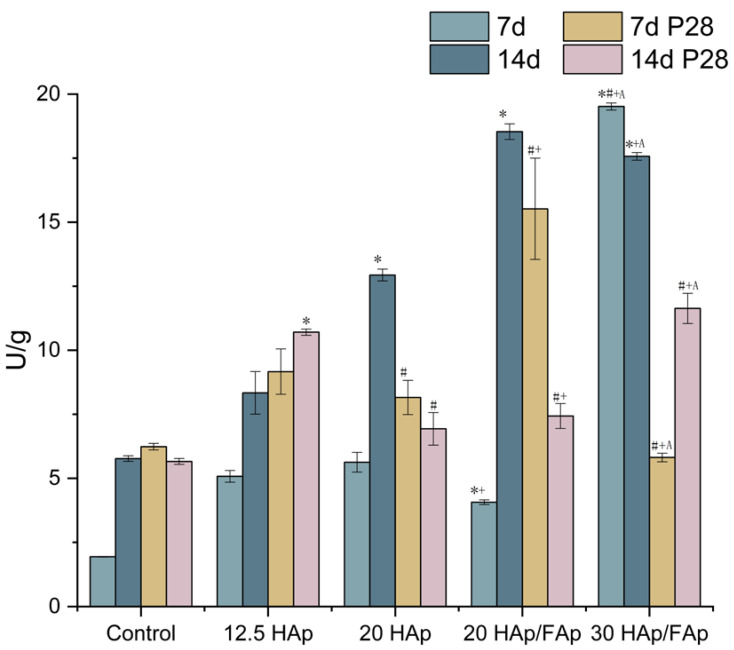
The quantification of alkaline phosphatase activity with different formulations of scaffolds and peptide-loaded scaffolds at 7 and 14 days. (* *p* < 0.05 vs. control, ^#^ *p* < 0.05 vs. 12 HAp, ^+^ *p* < 0.05 vs. 20 HAp, ^A^ *p* < 0.05 vs. 20 HAp/Fap).

The C2C12 cells were lysed at 7 and 14 days to determine the ALP activity, as shown in Figure 7. The cells secrete alkaline phosphatase, which is one of the characteristic parameters at the early osteoblastic differentiation stage [46]. The alkaline phosphatase activity determination proved to be a very useful indicator of the biomaterials’ ability to induce biomineralization [38,41,47].

The cell control group had a lower ALP expression compared to the 20 HAp and 30 HAp/FAp groups at 14 days. The ALP activities of the 20 HAp/FAp group were both significantly higher compared to those of the 20 HAp group at 7 and 14 days (*p* < 0.05). There was a significant increase in ALP activities between 7 and 14 days, which was observed in only the cell control group, 20 HAp group and 20 HAp/FAp group. Figure 7 demonstrates that ALP expression in the 12 HAp scaffolds after peptide loading was higher on days 7 and 14, respectively, compared to the nude 12 HAp scaffolds.

### 2.6. Quantitative Real-Time Polymerase Chain Reaction

We also evaluated the gene expression of osteoblast differentiation markers (*Runx2*, *Type I collagen* and *Osteocalcin*) at 7 and 14 days following co-culture with scaffolds and peptide-loaded scaffolds, by RT-qPCR. Differentiation was induced by scaffold and peptide-loaded scaffold in the C2C12 cells for 7 and 14 days. At both timepoints, we evaluated the expression of Runx2, Col1a1 and Bglap. We observed that the *Runx2* expression of each scaffold-treated group was increased at 7d in comparison to 14 d. This is expected, as *Runx2* is an early marker of osteogenic differentiation [48]. The positive control had increased expression at 14 d because the peptide was added when the medium changed. The early expression of *Runx2* is in line with the known differentiation path and indicates that the scaffolds have the ability to induce the cell differentiation [49].

**Figure 8:**
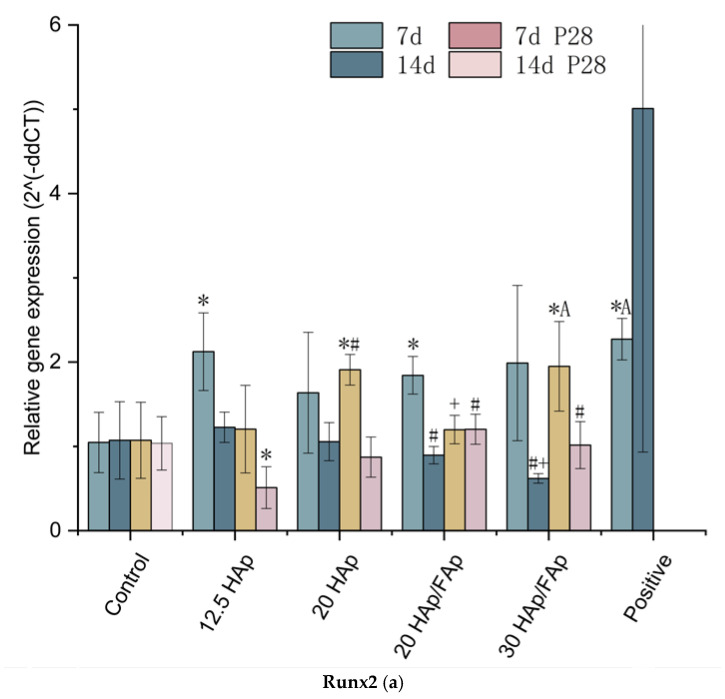

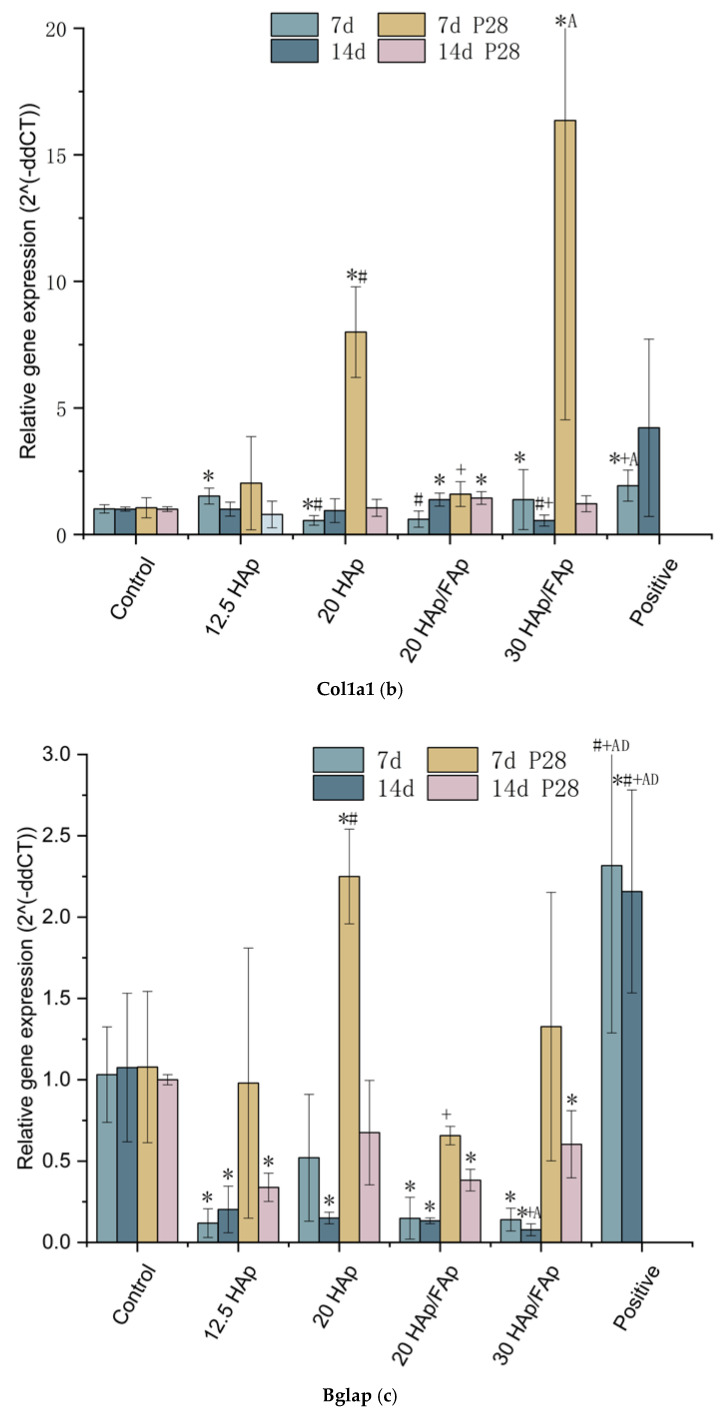
Relative levels of bone-associated RNAs for *Runx2* (**a**), *Col1a1* (**b**), and *Bglap* (**c**) from C2C12 cells seeded onto hybrid scaffold substrates and incubated for 7 and 14 days in the presence or absence of the peptide. (* *p* < 0.05 vs. control, ^#^ *p* <0.05 vs. 12 HAp, ^+^ *p* <0.05 vs. 20 HAp, ^A^ *p* <0.05 vs. 20 HAp/FAp, ^D^ *p* < 0.05 vs. 30 HAp/Fap).

*Cola1a* and *Bglap* are expected to be up-regulated at later stages of cellular differentiation [50]. However, we observed that the highest relative expression values for these genes were at the 7 d treatment with the P28-loaded scaffolds. We hypothesize that this is due the fact that the peptide is promptly released from the scaffold and accelerates the late stages of differentiation. Comparing P28-loaded scaffolds with the nude scaffold groups suggests the potential of the P28 peptide as an inducer in osteogenic differentiation, contributing to the bone matrix deposition (Figure 8). The 20 HAp group showed no significant up-regulation of the Col1a1 and Bglap genes at 7 d, compared to the non-peptide scaffold groups (*p* > 0.05).

## 3. Discussion

An ideal BTE scaffold should not only mimic the structure of natural bone but also support cell attachment, proliferation, and migration, as well as have antibacterial properties [17,26,51]. Furthermore, the osteogenic induction property is also important for the bone regeneration process. Therefore, one promising approach is to develop a biomimetic scaffold loaded with biological factors for induced bone regeneration [23,52]. BMP 2 has been applied in clinical treatment due to its ability to promote bone healing. However, BMP 2 still presents some drawbacks associated with the dose, carrier, and delivery approach. The P28 peptide is a promising derivative of BMP-2 which has the potential to overcome the drawbacks of BMP 2, while maintaining its functional advantages [26,45,49]. In the present study, the porous hybrid scaffolds were prepared through ice-sublimed freeze-drying. As a carrier for the P28 peptide, the system includes chitosan, hydroxyapatite, and fluorapatite. Many studies have focused on the CS/HAp bone scaffold because of its good biocompatibility, bone conductivity, and bioactivity. However, research has also shown the potential of FAp in BTE. Borkowski et al. reported that the appropriate combination of fluoride content leads to the synthesis of scaffolds with the best porosity, ion adsorption capacity, biological activity, and cell compatibility [22]. Fluorine ions may affect the physicochemical properties of bone tissue and the biological function of bone cells and can promote the crystallization of calcium phosphate and mineralization in bone formation [38,53].

In this work, the effect of the volume of CS was examined, along with the effect of HAp and FAp concentrations. It should be noted that although the naming of the samples corresponds to the AA concentration, this had an inverse effect on CS concentrations in the scaffolds, with lower AA volumes resulting in a higher concentration of CS in the final construct [54,55,56]. 

The scaffolds were evaluated by a SEM, which showed that the surface of the scaffolds was rough due to the ceramic particles present, providing sites for cell attachment [57]. Moreover, the scaffolds also exhibited interconnected macroporous microstructures that create an ideal proliferation environment for cells and facilitate the transport of nutrients, as well as metabolite removal [58,59].

The success of bone regeneration is sometimes limited by bacterial infections. Therefore, the antibacterial infection property of the bone scaffold is also important in BTE [60]. The bacteria that are most commonly associated with bone infections, or osteomyelitis, are typically Gram-positive. For example, Staphylococcus aureus is a Gram-positive bacterium and is one of the most frequent causes of bone-related infections [61,62]. The antibacterial properties of scaffolds showed the significant inhibition of Gram-positive bacteria (*S. aureus*) growth over 24 h (*p* < 0.05). As the volume of acetic acid increased, the increased protonation of amino groups in chitosan positively affected bacterial growth inhibition. The 20 HAp group had a lower bacterial proliferation rate compared to the 12 HAp group [63,64]. The addition of higher volumes of acetic acid may have caused an increase in the ions from the alkaline ceramic phase of the scaffold or, potentially, the increased acetic acid portion may have created a smoother surface, reducing the anchoring sites for bacteria [65]. Additionally, an increase in the ratio of FAp had a significantly positive effect on inhibiting bacterial growth due to the fact that fluoride ions directly restrain bacteria adhesion. This caused the 20 HAp/FAp to exhibit better antibacterial properties than 20 HAp (*p* < 0.05) [39,66].

Furthermore, the biological properties of scaffolds in vitro were investigated using the MTT experiments. These experiments indicated the viability of living cells co-cultured with a scaffold after 24 h. Compared with the control group, all scaffold co-cultured groups showed over 70% cell viability, revealing that scaffolds offered an ideal environment for cell proliferation [59,67].

The osteogenic differentiation properties of scaffolds were measured by the level of calcium deposition, alkaline phosphatase activity, and the expression of osteogenic genes [68]. These results showed that the ALP activity of scaffold groups was significantly higher compared to the cell-only group. The amounts of calcium deposition were significantly increased after being co-cultured with scaffolds. Additionally, the ALP activity of the P28-loaded scaffold significantly decreased compared to the nude scaffold groups (*p* < 0.05). However, the level of calcium mineral deposition increased as the P28 loaded on scaffolds. Moreover, the nude scaffolds also enhanced the osteogenic gene expression levels of C2C12 cells. It assumes that the scaffold material likely provides an appropriate growth environment for cell proliferation, and Ca^2+^ may induce osteoblast differentiation [69]. *Runx2* represents a key transcription factor during the early stage of osteoblast differentiation. The expression peak of *Runx2* normally presents at the preosteoblast/immature osteoblast stage, while it decreases in mature osteoblasts [23,70]. The collagen type I (*Col1a1*) expression is critical for osteoblast differentiation due to its role in forming the extracellular matrix. Furthermore, the upregulation of *Col1a1* expression is a marker of osteoblast maturation [68,71]. The Col1a1 expression of the 30 HAp/FAp group sharply increased after the P28 peptide was loaded. Finally, *Bglap* (osteocalcin), a late marker of osteogenesis, was shown to be up-regulated after the peptide was loaded to 20 HAp scaffolds compared with 20 HAp/FAp scaffolds at 7 days. The 30 HAp/FAp scaffolds showed up-regulation compared with the 20 HAp/FAp scaffolds as well [72,73]. We hypothesized that the burst release of the P28 peptide from the scaffold resulted in the *Bglap* expression being higher at 7 days. The hybrid scaffolds incorporated with the P28 peptide provide a promising approach for promoting bone regeneration and have stimulatory effects on matrix mineralization and osteoblastic cell differentiation [26].

This study does present some limitations. Firstly, we did not perform an in vitro study of P28 peptide release or establish concentration gradients to determine the most appropriate concentration for stimulating osteogenesis. However, we based our choice of P28 peptide concentration on the published papers of other researchers [49,74]. In addition, the current study focuses on the effect of P28-loaded hybrid scaffolds on the in vitro osteogenic differentiation of C2C12 cells. Future work will explore how the P28 peptide-loaded hybrid scaffold affects vascularization during the bone regeneration process.

## 4. Materials and Methods

High molecular chitosan (CS), acetic acid, hydroxyapatite, and sodium fluoride were purchased from Sigma-Aldrich (Wicklow, Ireland). The Purelink RNA Mini Kit, syber green master mix, and primers were obtained from ThermoFisher Bioscientific (Dublin, Ireland). The alkaline phosphatase assay kit and 1% Triton were obtained from Beyotime (Shanghai, China). P28 peptide was purchased from Pepmic (Suzhou, China). Poly (ethylene glycol) dimethacrylate (PEGDMA) was supplied by PolySciences Inc. (Polysciences Europe GmbH, Hirschberg an der Bergstrasse, Germany). The C2C12 murine myoblast cell line was purchased from the European Collection of Cell Cultures (ECACCs). MACSQuant^®^ washing solution and running buffer were purchased from Miltenyi Biotec (Bisley, UK). All other reagents used in the laboratory were of the highest quality available commercially.

### 4.1. Scaffold Preparation

The CS/HAp/FAp scaffolds were prepared by freeze-drying. Scaffolds were prepared as follows: The ceramic was dissolved in ultrapure water and stirred for 1 h. The CS powder was used to prepare different yield pastes with 1% acetic acid for 1 h. Then, sodium bicarbonate was added to the paste as neutralization. The obtained paste was added to 100 µL PEGDMA and 10 µL 0.1% *w/v* benzophenone solution in ethanol. Then, the ceramic solution was added to the CS paste and mixed thoroughly. The obtained paste was placed into a cylindrical silicon mold containing 10 circular impressions (25 mm dia, height 5 mm) and UV-irradiated to initiate the cross-linking reaction. The wavelength was between 315 and 400 nm, at an average intensity of 10–13.5 mW cm^2^. Scaffolds were cured for 40 min turning the sample over mid-curing (as shown in Table 1). The scaffolds were collected after UV irradiation and transferred to a Petri dish to freeze at −80 °C overnight. The scaffolds were freeze-dried by LyoLab 3000 Freeze Dryer (Thermo Scientific, UK).

### 4.2. Peptide Loading

The freeze-dried scaffolds were sterilized for 30 min in a UV chamber in advance. P28 peptide (S[PO4]DDDDDDDKIPKASSVPTELSAISTLYL, molecular weight: 3091.20) was dissolved in 500 μL of medium and mixed well. P28-loaded scaffolds were prepared by dropping a P28 solution (10 mg/mL) onto the scaffolds under sterile conditions, allowing it to be absorbed. Finally, P28-loaded scaffolds were stored at −20 °C for later use.

### 4.3. Scanning Electron Microscopy (SEM)

The morphology of the scaffolds was observed using a SEM. The scaffolds were sputtered with a gold coating and placed on an aluminum stub (0.1 mBar vacuum, Baltec SCD 005). Three random visual fields were selected for evaluating the ceramic distribution of the surface morphology of scaffolds.

### 4.4. Antibacterial Test

The hybrid scaffolds were used with molds having a diameter of 4.8 mm and a height of 1.7 mm which were sterilized by UV light radiation for 30 min. The *Staphylococcus aureus* was cultured in nutrient broth and incubated at 37 °C and 100 rpm for 24 h. The bacterial inoculum was prepared by diluting the 24 h culture with nutrient broth to a concentration of 1 × 10^4^ CFU/mL; 100 µL of bacteria suspension was transferred onto the scaffold surface in 24 well plates and incubated for 24 h at 37 °C. This volume was enough to cover the scaffold. A 100 µL sample was collected after mixing by pipetting up and down 10× and centrifuged at 9000 rpm for 5 min. The supernatant was discarded and, in order to wash the bacterial pellet, a volume of 500 µL of PBS was added, and the solution was vortex for 5 min. Samples were centrifuged at 9000 rpm for 5 min, and this process was repeated twice before incubating the washed pellet with 200 µL of SYBR™ Green I (Invitrogen) for 40 min in the dark to verify cell viability. This dye was chosen based on its ability to bind nucleic acids without affecting damaged/dead bacterial cells. After incubation, samples were centrifuged at 9000 rpm for 5 min and the supernatant was discarded. Finally, stained bacterial pellets were resuspended in 500 µL of running buffer (MACSQuant^®^), and 200 µL was transferred to 96 well plates, and cell viability was evaluated by flow cytometry (MACSQuant^®^ Analyzer 10 Flow Cytometer, Miltenyi Biotec, Bisley, UK). The gating strategy was based on 3 steps: first, cells were selected (side scatter × forward scatter), singlets were separated from doublets and clusters (area scatter × forward scatter), and viable cells emitting green fluorescence from SYBR™ Green I stain were verified in the channel FITC-A (FITC-A × forward scatter). A negative control (bacteria incubated in ethanol for 15 min), non-stained control (200 µL of PBS added instead of SYBR™ Green I), and positive control (bacteria in broth stained with SYBR™ Green I) were also prepared to assist in gating viable cells.

### 4.5. Cell Seeding

C2C12 cells, a mouse myoblast cell line, were cultured in Dulbecco’s Modified Eagle Medium (DMEM), supplemented with 10% Fetal Bovine Serum (FBS), 1% of L-Glutamine, and 1% of penicillin/streptomycin. The cells were maintained at 37 °C, 5% CO2 in a humid incubator. The cells were passaged when approaching 80% confluency, and 4 × 10^4^/well cells were seeded in each well in a 12-well plate for the Alizarin Red assay and for RNA isolation, and in each well of a 96-well plate for the MTT assay.

### 4.6. Cytotoxicity Assay

The viability of C2C12 cells on the scaffolds was analyzed using the MTT (3-[4,5-dimethylthiazol-2-yl]-2,5-diphenyltetrazolium bromide) assay. The principle of the assay is based on the reduction of the tetrazolium ring of MTT by living cells, thus indicating cell viability. The scaffolds were immersed into DMEM overnight for preparing eluate in DMEM. The cells were seeded as described before. The C2C12 cells were incubated overnight with scaffolds eluate in DMEM using 96-well plates. The cells were then washed with a phosphate buffer saline, and 100 μL of MTT solution (5 mg/mL) was added to each well. The cells were incubated for 4 h at 37 °C in CO2, protected from light. After incubation, the medium was discarded, and the formazan crystals formed were dissolved in DMSO. Absorbance was read at 570 nm using a microplate reader (Perkin Elmer, Waltham, MA, USA).

### 4.7. Alizarin Red S Staining

Alizarin Red S (ARS) staining was assessed for the presence of calcium deposition of cells. After 7 d and 14 d of incubation, the medium was removed and washed with PBS two times. Then, the cells were fixed by treatment with 4% formaldehyde for 15 min. After fixation, the cells were stained with 40 mM Alizarin Red for 20 min at room temperature and then washed with distilled water. The stained cells were photographed under a microscope equipped with a camera. 

The dried stained cells were cultured with 10% acetic acid for 30 min. After being detached, the cells were transferred into a 1.5 mL Eppendorf tube. Then, the tubes were heated in a water bath at 85 °C for 10 min; centrifuged at 2000× *g* for 15 min; and transferred to ice for 5 min. Next, the supernatant was transferred to new tube with 10% ammonium hydroxide to neutralize the acidic environment. The solubilized calcium-bound Alizarin Red S was determined by the plate reader (Perkin Elmer, USA) at 405 nm.

### 4.8. Alkaline Phosphatase (ALP) Activity

The scaffolds were analyzed for the effect of C2C12 cells by quantitative measurement of alkaline phosphatase (ALP), which is an unequivocal marker of osteogenic differentiation at an early stage. The suspension of C2C12 cells was seeded and incubated with the scaffolds as mentioned earlier. At 7 and 14 days, the cells were washed three times with PBS after the medium and scaffolds were removed. Then, the cells were lysed using 1% Triton X-100 at 4 °C for about 15 min. To examine the ALP activity of C2C12 cells by pNPP-Na, we conducted a quantitative analysis of ALP, following the manufacturer’s instructions (Beyotime, Shanghai, China). Subsequently, the optical density was measured at 405 nm using a Multilabel Counter Wallac 1420 (Perkin Elmer, USA).

### 4.9. Quantitative Real-Time Polymerase Chain Reaction

Total RNA was isolated from C2C12 cells on 7 and 14 d using Purelink RNA Mini Kit (Thermo Fisher Sciencentific, Waltham, MA, USA), following the manufacturer’s protocol. RNA was quantitatively assessed by QubitTM 3 fluorometer and the RNA concentration was standardized for all samples prior to cDNA synthesis. cDNA synthesis was performed with the High Capacity cDNA Kit (ThermoFisher Scientific) with the use of RNAse inhibitor, following manufacturer’s instructions, and stored at −20 °C for later use.

Gene expression was evaluated by real time RT-qPCR with the PowerUp™ SYBR™ Green Master Mix (ThermoFisher Scientific), and each reaction consisted of 5 μL of the master mix; 3.5 μL of molecular grade water; 0.25 μL of each primer (forward and reverse) from a 10 μM stock; and 1 μL of cDNA (template). The reaction was performed with the 7300 Real Time PCR System (Applied Biosystems, Waltham, MA, USA), using the “standard 7300” run mode and the following settings: 1 cycle at 50 °C for 2 min; 1 cycle at 95 °C for 2 min; 45 cycles at 95 °C for 15 s, 60 °C for 15 s, and 72 °C for 1 min, followed by a dissociation stage of 95 °C for 15 s, 60 °C for 1 min, 95 °C for 15 s, and 60 °C for 15 s. Tbp was used as a housekeeping gene, and Bglap, CoI1a1, and Runx2 were used as specific markers of osteoblast cells. All the sequences of primers are shown in Table 2.

### 4.10. Statistical Analysis

The results were obtained from triplicate samples and presented as means ± standard deviations. The significance of difference was carried out by one-way analysis of variance (ANOVA) with Minitab. *p* ≤ 0.05 was considered statistically significant.

## 5. Conclusions

In summary, we successfully synthesized a CS/ceramic hybrid scaffold by UV crosslinking and freeze-drying methods. The hybrid scaffolds mimic the natural bone structure, with a high percentage of interconnected porosity, which was assessed by a SEM. The in vitro cell toxicity test results showed that the hybrid scaffolds can promote cell attachment, viability, and proliferation. Furthermore, these results also demonstrated that the peptide-loaded scaffolds up-regulated the expression of osteogenic genes at both the early and late stages of osteogenic differentiation after 7 and 14 days of co-culture with C2C12 cells. On the other hand, they showed that peptide-loaded scaffolds preformed better in accelerating osteogenic expression. The hybrid scaffolds showed promising potential for promoting the repair of large bone defects.

## Figures and Tables

**Table 1:** The formulations of hybrid scaffolds.

Scaffold ID	CS (g)	HAp (g)	FAp (g)	Volume of Acetic Acid/(mL)
12.5 HAp	1.5	1.5	0	12.5
20 HAp	1.5	1.5	0	20
20 HAp/FAp	1.5	0.75	0.75	20
30 HAp/FAp	1.5	0.75	0.75	30

**Table 2:** Primer sequences used for real-time RT-PCR.

Gene	Primer Sequences Forward/Reverse
*Bglap*	5′-GACACCATGAGGACCATCTTTC-3′/5′-CATGAAGGCTTTGTCAGACTCA-3′
*Col1a1*	5′-CCAATGGTGCTCCTGGTATT-3′/5′-GGTTCACCACTGTTACCCTT-3′
*Runx2*	5′-CTCTGATCGCCTCAGTGATTT-3′/5′-CTGCCTGGGATCTGTAATCTG-3′
*Tbp*	5′-AGTGCCCAGCATCACTATTT-3′/5′-GGTCCATGATTCTCCCTTTCTT-3′

## Data Availability

Data are contained within the article.
